# Supplementary material for: Specific Glucagon Assay System Using a Receptor-Derived Glucagon-Binding Peptide Probe
Source: Int J Mol Sci. 2026 Jan 4;27(1):515. doi: 10.3390/ijms27010515 (PMC12786957; doi:10.3390/ijms27010515)
Supplement: Supplementary file 1 [file ijms-27-00515-s001.zip › ijms-4036468-supplementary.pdf]

Supplementary Materials

Specific Glucagon Assay System Using a Receptor-Derived Glucagon-Binding Peptide Probe

Hajime Shigeto [1,*], Yoshio Suzuki [2] and Shohei Yamamura [1,*]

[1] Health and Medical Research Institute, National Institute of Advanced Industrial Science and Technology (AIST), 2217-14 Hayashi-cho, Takamatsu 761-0395, Japan
[2] Health and Medical Research Institute, National Institute of Advanced Industrial Science and Technology (AIST), Central 6, 1-1-1 Higashi, Tsukuba 305-8566, Japan; suzuki-yoshio@aist.go.jp
* Correspondence: hajime.shigeto@aist.go.jp (H.S.); yamamura-s@aist.go.jp (S.Y.)

1	2	3	4	5	6
7	8	9	10	11	12
13	14	15	16	17	18
19	20	21	22	23	24
25	26	27	28	29	30
31	32	33	34	35	36
37	38	39	40	41	42
43	44	45	46	47	48
49	50	51	52	53	54
55	56	57	58	59	60
61	62	63	64	65	66
67	68	69	70	71	72
73	74	75	76	77	78
79	80	81	82	83	84
85	86	87	88	89	90
91	92	93	94		

1	MPPCQPQRPLLLLLL	34	LSKLHCTRNAIHANL	67	AKLRARQMHHTDYKF
2	PQRPLLLLLLLLACQ	35	CTRNAIHANLFASFV	68	RQMHHTDYKFRLAKS
3	LLLLLLLACQPQVPS	36	IHANLFASFVLKASS	69	TDYKFRLAKSTLTLI
4	LLACQPQVPSAQVMD	37	FASFVLKASSVLVID	70	RLAKSTLTLIPLLGV
5	PQVPSAQVMDFLFEK	38	LKASSVLVIDGLLRT	71	TLTLIPLLGVHEVVF
6	AQVMDFLFEKWKLYG	39	VLVIDGLLRTRYSQK	72	PLLGVHEVVFAFVTD
7	FLFEKWKLYGDQCHH	40	GLLRTRYSQKIGDDL	73	HEVVFAFVTDEHAQG
8	WKLYGDQCHHNLSLL	41	RYSQKIGDDLSVSTW	74	AFVTDEHAQGTLRSA
9	DQCHHNLSLLPPPTE	42	IGDDLSVSTWLSDGA	75	EHAQGTLRSAKLFFD
10	NLSLLPPPTELVCNR	43	SVSTWLSDGAVAGCR	76	TLRSAKLFFDLFLSS
11	PPPTELVCNRTFDKY	44	LSDGAVAGCRVAAVF	77	KLFFDLFLSSFQGLL
12	LVCNRTFDKYSCWPD	45	VAGCRVAAVFMQYGI	78	LFLSSFQGLLVAVLY
13	TFDKYSCWPDTPANT	46	VAAVFMQYGIVANYC	79	FQGLLVAVLYCFLNK
14	SCWPDTPANTTANIS	47	MQYGIVANYCWLLVE	80	VAVLYCFLNKEVQSE
15	TPANTTANISCPWYL	48	VANYCWLLVEGLYLH	81	CFLNKEVQSELRRRW
16	TANISCPWYLPWHHK	49	WLLVEGLYLHNLLGL	82	EVQSELRRRWHRWRL
17	CPWYLPWHHKVQHRF	50	GLYLHNLLGLATLPE	83	LRRRWHRWRLGKVLW
18	PWHHKVQHRFVFKRC	51	NLLGLATLPERSFFS	84	HRWRLGKVLWEERNT
19	VQHRFVFKRCGPDGQ	52	ATLPERSFFSLYLGI	85	GKVLWEERNTSNHRA
20	VFKRCGPDGQWVRGP	53	RSFFSLYLGIGWGAP	86	EERNTSNHRASSSPG
21	GPDGQWVRGPRGQPW	54	LYLGIGWGAPMLFVV	87	SNHRASSSPGHGPPS
22	WVRGPRGQPWRDASQ	55	GWGAPMLFVVPWAVV	88	SSSPGHGPPSKELQF
23	RGQPWRDASQCQMDG	56	MLFVVPWAVVKCLFE	89	HGPPSKELQFGRGGG
24	RDASQCQMDGEEIEV	57	PWAVVKCLFENVQCW	90	KELQFGRGGGSQDSS
25	CQMDGEEIEVQKEVA	58	KCLFENVQCWTSNDN	91	GRGGGSQDSSAETPL
26	EEIEVQKEVAKMYSS	59	NVQCWTSNDNMGFWW	92	SQDSSAETPLAGGLP
27	QKEVAKMYSSFQVMY	60	TSNDNMGFWWILRFP	93	AETPLAGGLPRLAES
28	KMYSSFQVMYTVGYS	61	MGFWWILRFPVFLAI	94	TPLAGGLPRLAESPF
29	FQVMYTVGYSLSLGA	62	ILRFPVFLAILINFF		
30	TVGYSLSLGALLLAL	63	VFLAILINFFIFVRI		
31	LSLGALLLALAILGG	64	LINFFIFVRIVQLLV		
32	LLLALAILGGLSKLH	65	IFVRIVQLLVAKLRA		
33	AILGGLSKLHCTRNA	66	VQLLVAKLRARQMHH		

Figure. S1

Amino-acid sequences in peptide array used for screening glucagon-binding peptides from the glucagon receptor sequence.

No.15

Oxyntomodulin

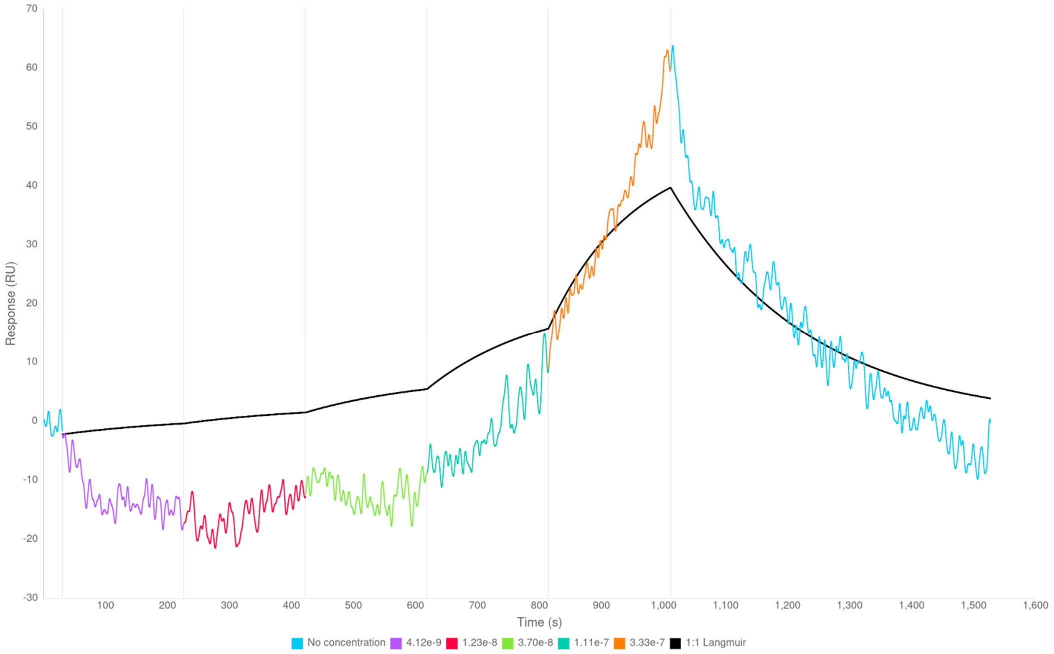

Mini-Glucagon

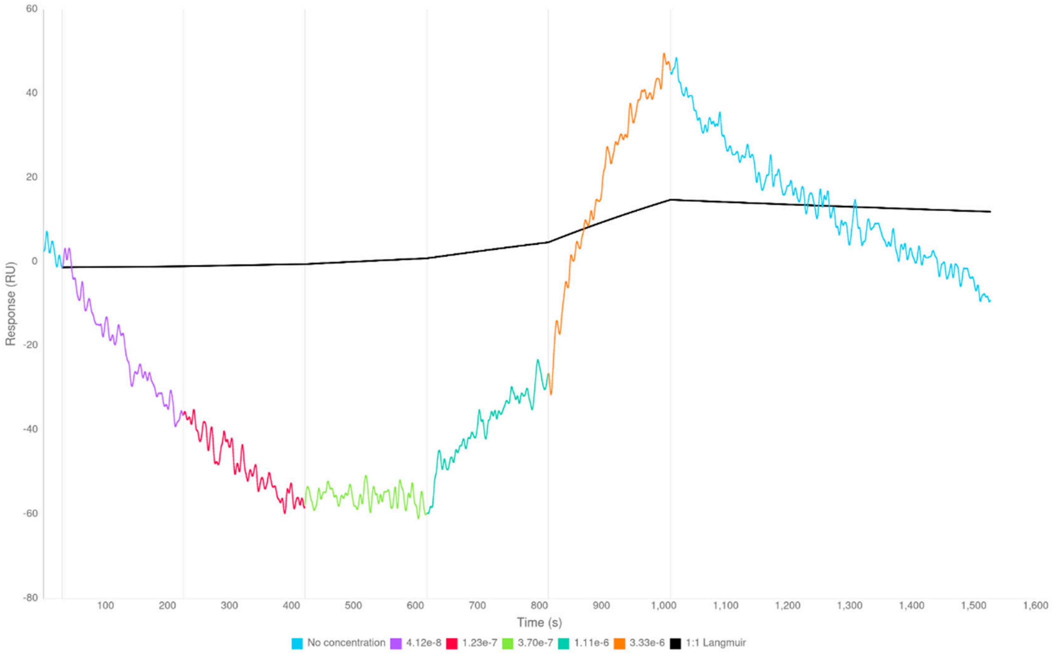

Glicentin

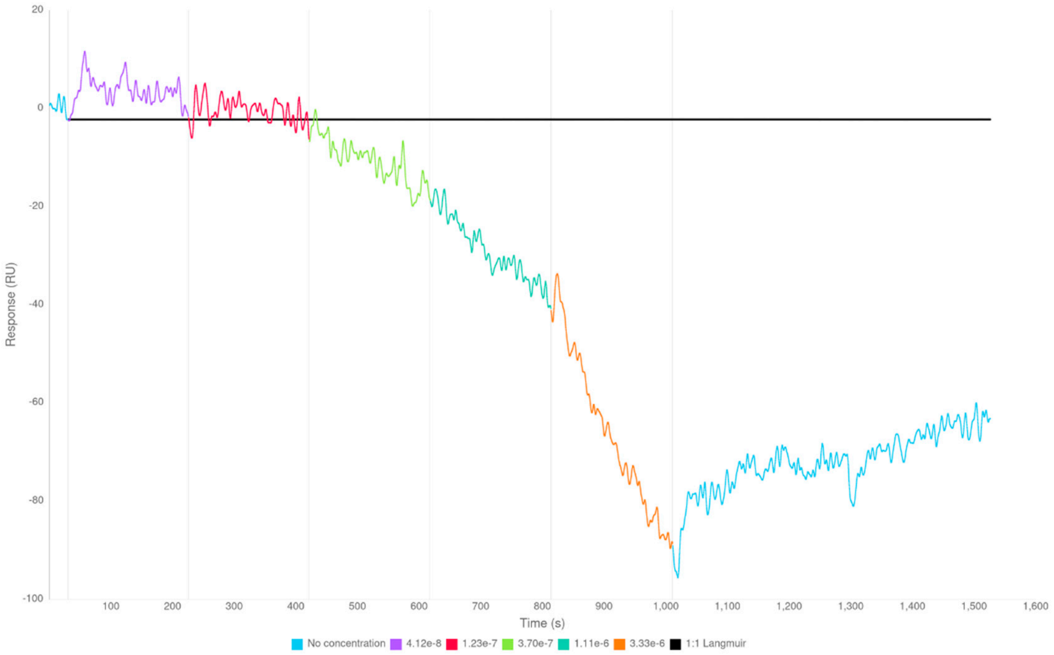

No.32

Oxyntomodulin

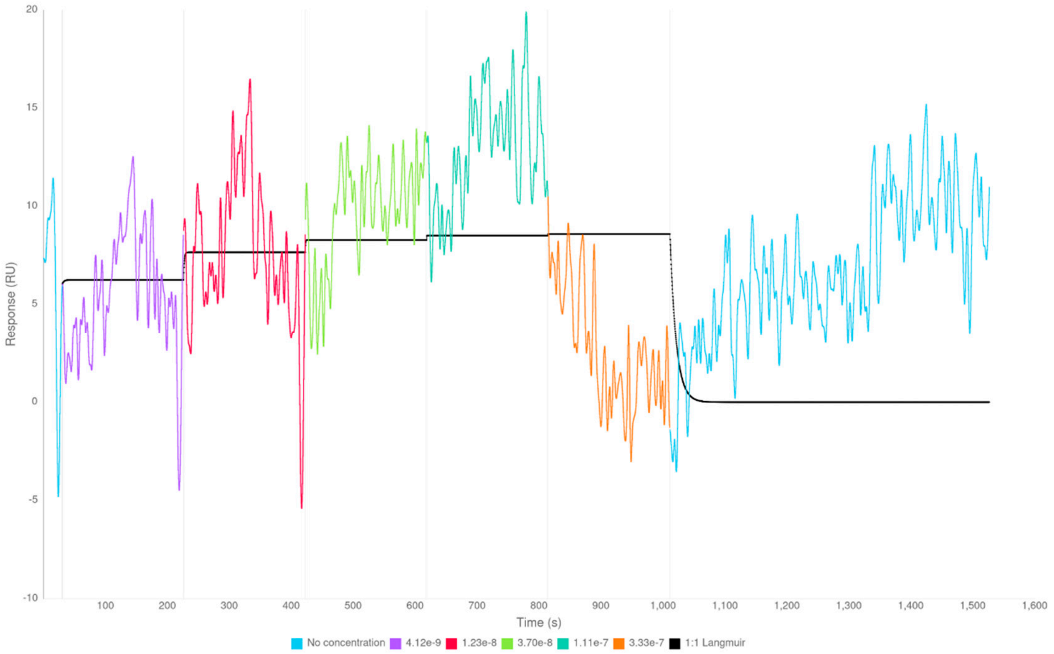

Mini-Glucagon

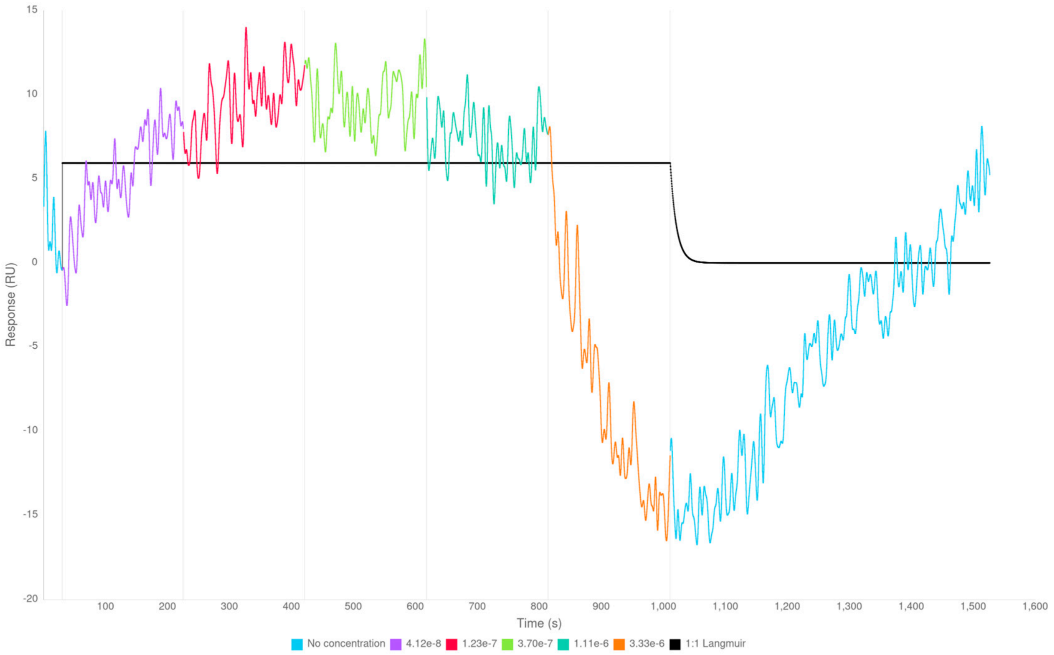

Glicentin

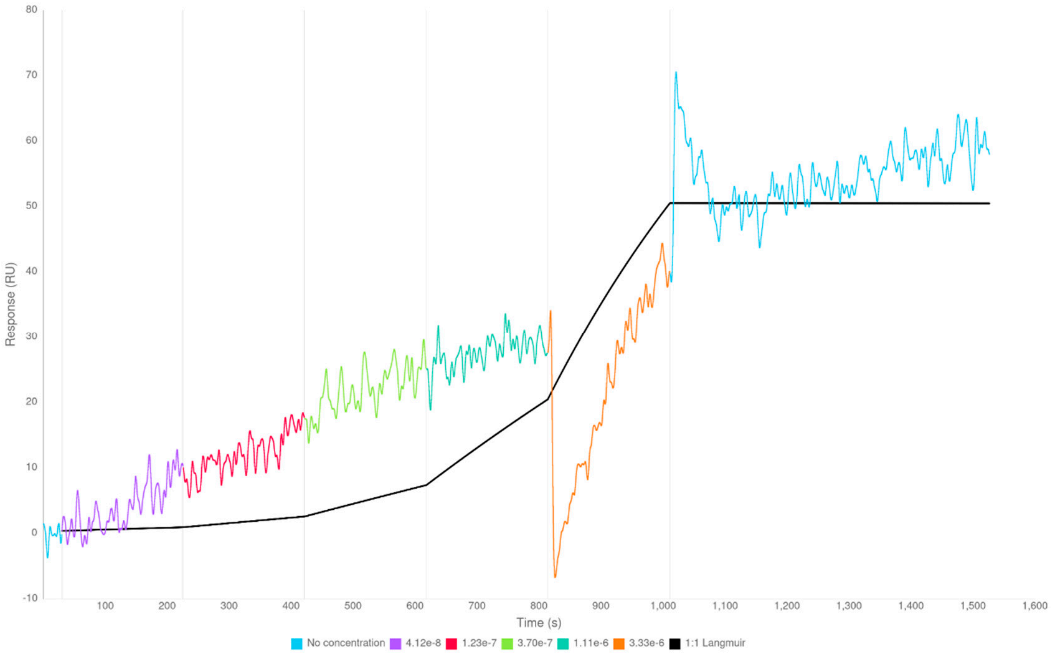

No.59

Oxyntomodulin

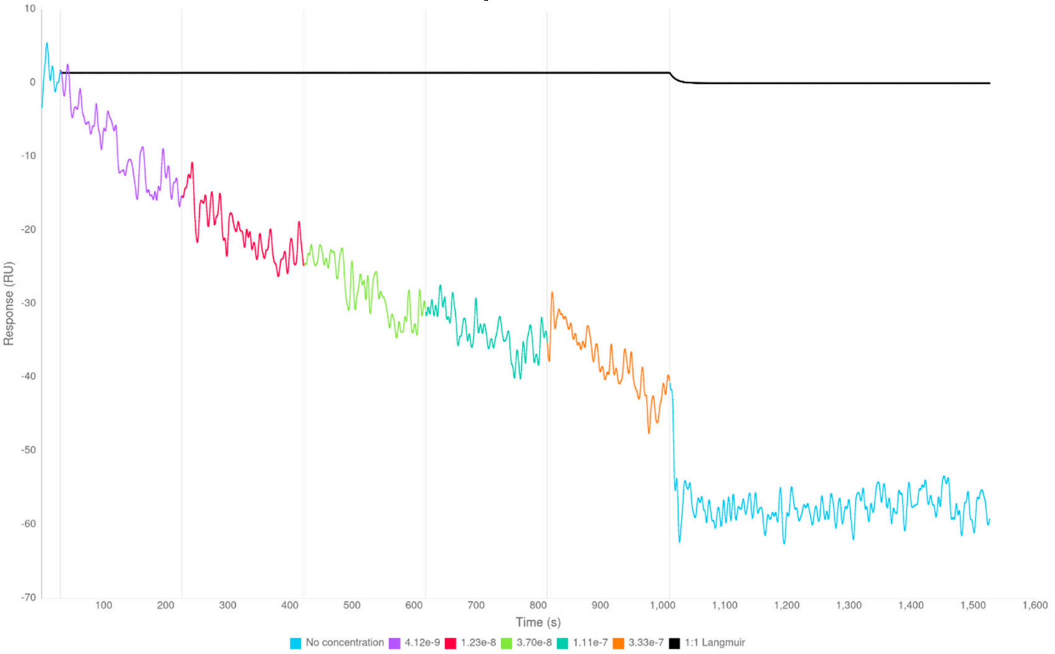

Mini-Glucagon

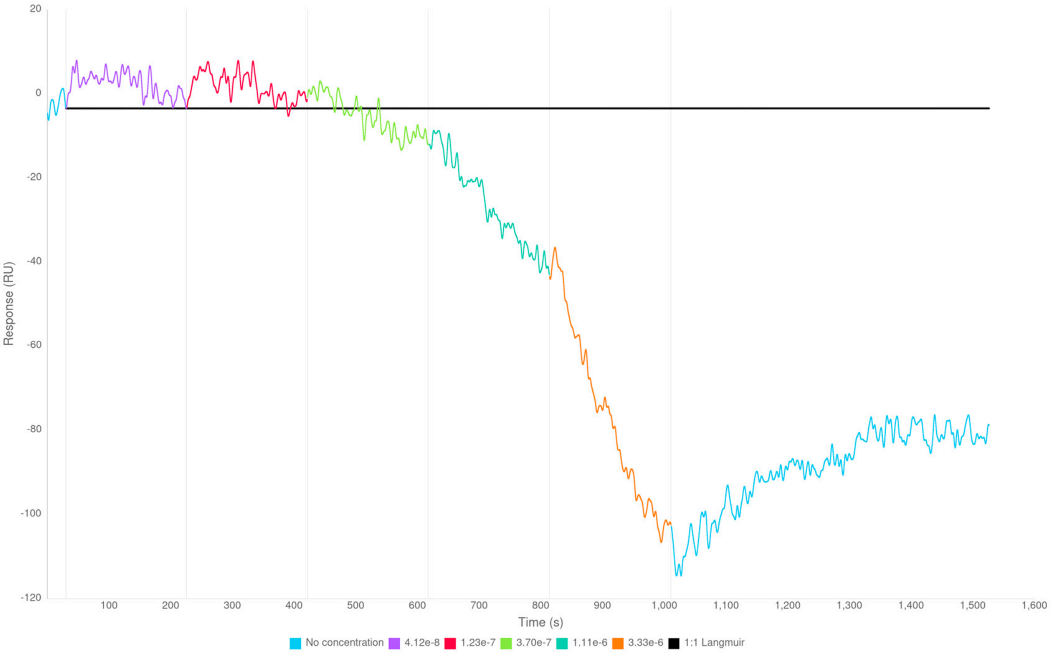

Glicentin

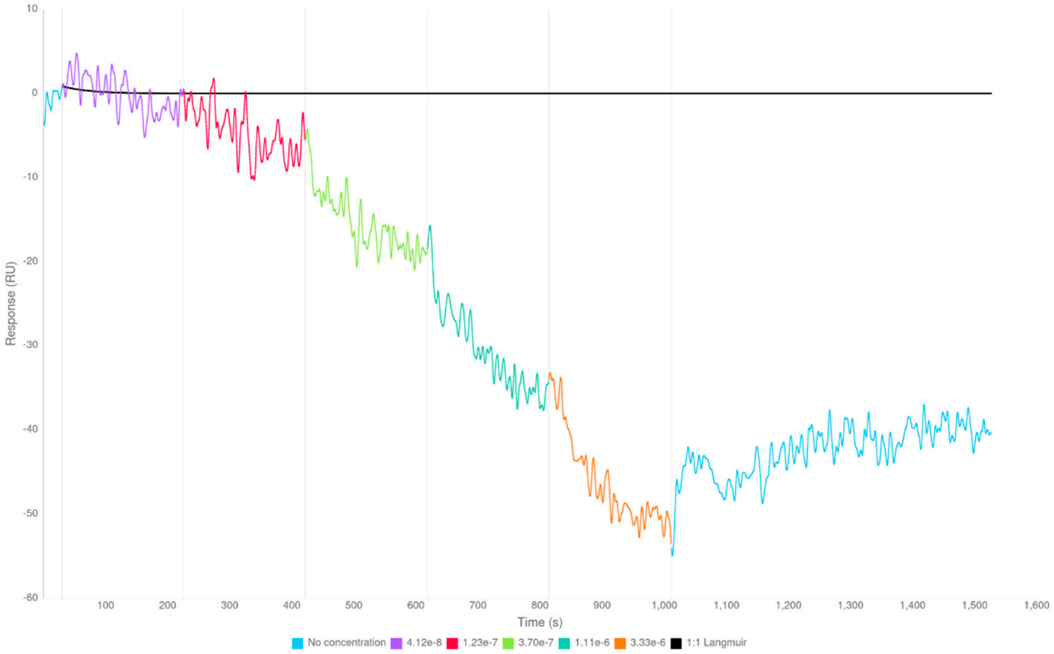

No.65

Oxyntomodulin

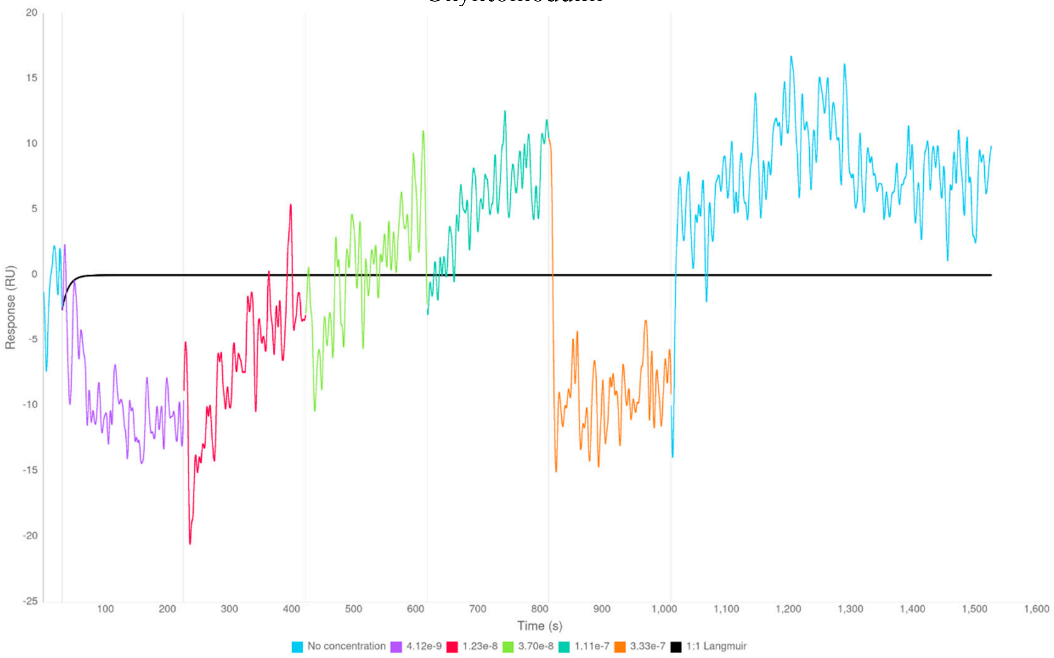

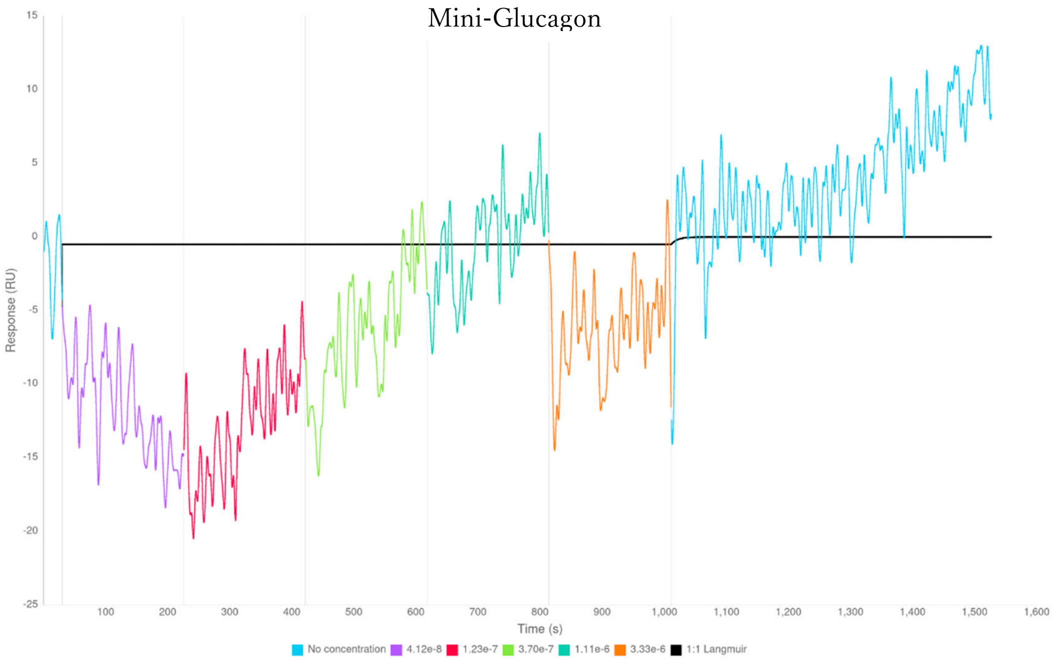
Mini-Glucagon
Response (RU)
Time (s)
No concentration
4.12e-8
1.23e-7
3.70e-7
1.11e-6
3.33e-6
1:1 Langmuir

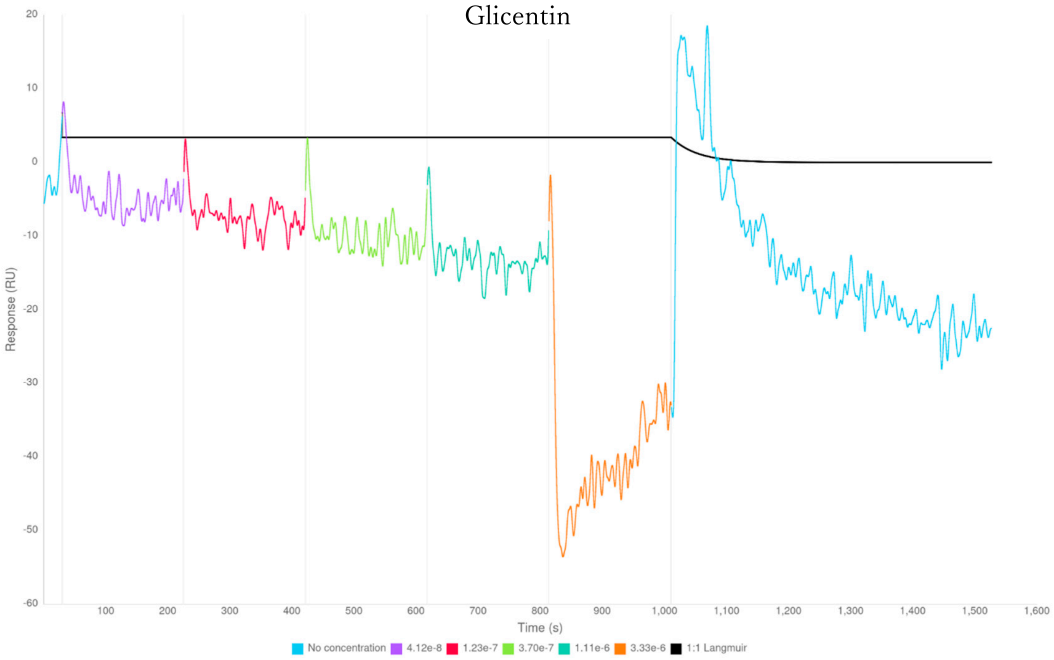
Glicentin
Response (RU)
Time (s)
No concentration
4.12e-8
1.23e-7
3.70e-7
1.11e-6
3.33e-6
1:1 Langmuir

Figure. S2

Intramolecular functional analysis results of the screened peptides (No. 15, 32, 59, and 65) against glucagon analogs. Each biotinylated peptide was immobilized at the sensor surfaces of the reaction cartridge. Oxyntomodulin, mini-glucagon, and glicentin (1-100 μM) were gradually diluted automatically with 100 mM sodium acetate buffer (pH 5.2) containing 0.1% Tween 20 and reacted to immobilized peptides.

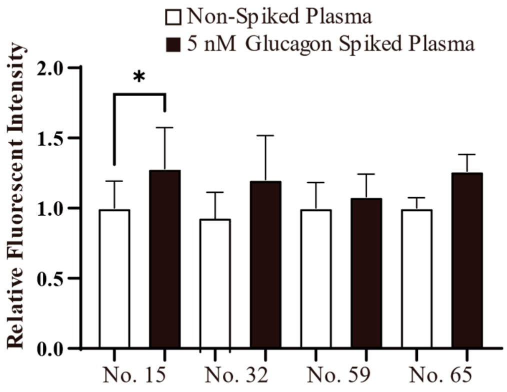

Figure. S3

Measurement of glucagon in human plasma using the GBPs (No.15, 32, 59, 65). Normal human whole blood was purchased (Central Link, Tokyo, Japan) and collected the plasma. The collected plasma was diluted ten-fold with H_2O. The glucagon was spiked in prepared sample and reacted with the GBPs. Relative intensities were normalized by the intensities of non-spiked plasma. Data are expressed as mean ± standard deviation of three replicates. *$P < 0.05$, one-way ANOVA followed by Tukey's comparison test.

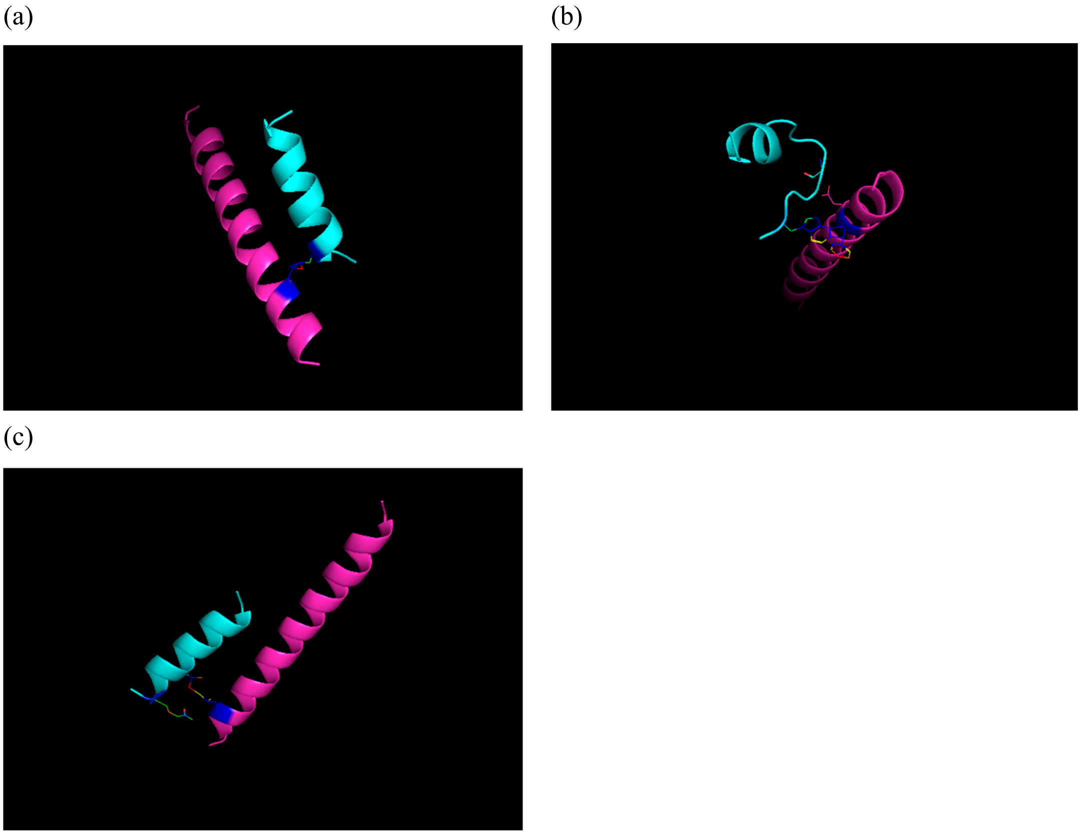

Figure. S4.

The binding hypothetical model of glucagon (pink) and each peptide (cyan) estimated by AlphaFold2. (a)The glucagon (pink) and peptide No. 32 peptide (cyan). The L3 side chains of peptide No. 32 and V23 side chains of glucagon are indicated. (b) The glucagon (pink) and peptide No. 59 (cyan). The W14 side chains of peptide No. 59 and F22 side chains of glucagon are indicated. (c) The glucagon (pink) and peptide No. 65 (cyan). The R14 side chains of peptide No. 65 and L26 side chains of glucagon are indicated.
